# Clinical and biological significance of *de novo* CD5^+^ diffuse large B-cell lymphoma in Western countries

**DOI:** 10.18632/oncotarget.3479

**Published:** 2015-03-08

**Authors:** Zijun Y. Xu-Monette, Meifeng Tu, Kausar J. Jabbar, Xin Cao, Alexandar Tzankov, Carlo Visco, Qingqing Cai, Santiago Montes-Moreno, Yuji An, Karen Dybkaer, April Chiu, Attilio Orazi, Youli Zu, Govind Bhagat, Kristy L. Richards, Eric D. Hsi, William W.L. Choi, J. Han van Krieken, Jooryung Huh, Maurilio Ponzoni, Andrés J.M. Ferreri, Xiaoying Zhao, Michael B. Møller, John P. Farnen, Jane N. Winter, Miguel A. Piris, Roberto N. Miranda, L. Jeffrey Medeiros, Ken H. Young

**Affiliations:** ^1^ Department of Hematopathology, The University of Texas MD Anderson Cancer Center, Houston, TX, USA; ^2^ Peking University Cancer Hospital and Institute, Beijing, China; ^3^ University Hospital, Basel, Switzerland; ^4^ San Bortolo Hospital, Vicenza, Italy; ^5^ Hospital Universitario Marques de Valdecilla, Santander, Spain; ^6^ Aalborg University Hospital, Aalborg, Denmark; ^7^ Memorial Sloan-Kettering Cancer Center, New York, NY, USA; ^8^ Weill Medical College of Cornell University, New York, NY, USA; ^9^ The Methodist Hospital, Houston, TX, USA; ^10^ Columbia University Medical Center and New York Presbyterian Hospital, New York, NY, USA; ^11^ University of North Carolina School of Medicine, Chapel Hill, NC, USA; ^12^ Cleveland Clinic, Cleveland, OH, USA; ^13^ University of Hong Kong Li Ka Shing Faculty of Medicine, Hong Kong, China; ^14^ Radboud University Nijmegen Medical Centre, Nijmegen, Netherlands; ^15^ Asan Medical Center, Ulsan University College of Medicine, Seoul, Korea; ^16^ San Raffaele H. Scientific Institute, Milan, Italy; ^17^ Zhejiang University School of Medicine, Second University Hospital, Hangzhou, China; ^18^ Odense University Hospital, Odense, Denmark; ^19^ Gundersen Lutheran Health System, La Crosse, WI, USA; ^20^ Feinberg School of Medicine, Northwestern University, Chicago, IL, USA; ^21^ The University of Texas School of Medicine, Graduate School of Biomedical Sciences, Houston, Texas, USA

**Keywords:** ABC, BCL2, CD5, diffuse large B-cell lymphoma, NF-κB

## Abstract

CD5 is a pan-T-cell surface marker and is rarely expressed in diffuse large B-cell lymphoma (DLBCL). Large-scale studies of *de novo* CD5^+^ DLBCL are lacking in Western countries. In this study by the DLBCL Rituximab-CHOP Consortium, CD5 was expressed in 5.5% of 879 DLBCL patients from Western countries. CD5^+^ DLBCL was associated with higher frequencies of >1 ECOG performance status, bone marrow involvement, central nervous system relapse, activated B-cell–like subtype, Bcl-2 overexpression, and STAT3 and NF-κB activation, whereas rarely expressed single-stranded DNA-binding protein 2 (SSBP2), CD30 or had *MYC* mutations. With standard R-CHOP chemotherapy, CD5^+^ DLBCL patients had significantly worse overall survival (median, 25.3 months *vs*. not reached, *P*< .0001) and progression-free survival (median, 21.3 vs. 85.8 months, *P*< .0001) than CD5^−^ DLBCL patients, which was independent of Bcl-2, STAT3, NF-κB and the International Prognostic Index. Interestingly, SSBP2 expression abolished the prognostic significance of CD5 expression, suggesting a tumor-suppressor role of SSBP2 for CD5 signaling. Gene-expression profiling demonstrated that B-cell receptor signaling dysfunction and microenvironment alterations are the important mechanisms underlying the clinical impact of CD5 expression. This study shows the distinctive clinical and biological features of CD5^+^ DLBCL patients in Western countries and underscores important pathways with therapeutic implications.

## INTRODUCTION

CD5 is a cell surface glycoprotein typically expressed on normal and neoplastic T- cells, as well as on a subset of normal naïve B-cells and lymphoma cells, mainly in chronic lymphocytic leukemia/small lymphocytic lymphoma (CLL/SLL) and mantle cell lymphoma [1-4]. CD5 has an immunoreceptor tyrosine inhibitory motif and functionally inhibits the T-cell response [5, 6] and B-cell receptor (BCR) signaling-mediated apoptosis, probably by recruiting the SH2 domain-containing protein tyrosine phosphatase-1 (SHP-1) after being phosphorylated by Lyn [7, 8]. In lymphocytes, CD5 also inhibits signaling downstream of the BCR pathway, including the calcium response and interleukin-2 (IL2) production whereas augments BCR-mediated IL10 production, an anti-inflammatory cytokine and a survival factor for B-cells [9,10]. In CLL/SLL, CD5 governs the phosphorylation and nuclear translocation of STAT3 and nuclear factor of activated T cells 2 (NFAT2); activated STAT3/NFAT2 in turn leads to excess production of IL10 [11, 12].

CD5 has also been found to be expressed, albeit rarely, in *de novo* diffuse large B-cell lymphoma (DLBCL). To date, large-scale studies of *de novo* CD5^+^ DLBCL have been conducted only in Japan, with a reported frequency of 5 to 22% of all DLBCL [13-20]. Compared with patients with CD5^−^ DLBCL, CD5^+^ DLBCL patients are reportedly more often elderly, female, and have >1 ECOG performance status, elevated serum lactate dehydrogenase (LDH) level, advanced stage disease, >1 extranodal sites, B-symptoms, and high International Prognostic Index (IPI) at diagnosis [13, 14, 19]. Pathologically, CD5^+^ DLBCL are associated with centroblastic morphology (rarely immunoblastic), Bcl-2 overexpression, and non-germinal center B-cell (non-GCB) subtype [16,19].

Most studies from Japan have shown that clinical outcomes of CD5^+^ DLBCL patients treated with standard CHOP (cyclophosphamide, doxorubicin, vincristine, and prednisone) chemotherapy with or without rituximab are poor, although the prognostic significance of CD5 positivity may depend on associated aggressive clinical parameters [13-15, 17, 19]. Bone marrow (BM) involvement (28%) and central nervous system (CNS) relapse (12.7%) are increased in CD5^+^ DLBCL patients [14, 16, 20]. However, in one study CD5 expression status did not correlate with prognosis by univariate or multivariate analysis, either in all patients or in rituximab-treated patients [18]. The effect of adding rituximab to CHOP on survival of CD5^+^ DLBCL patients also has been inconsistent in different studies [18-20]. In Western countries, a few cases of *de novo* CD5^+^ DLBCL have been reported [21, 22], and a morphologic and immunophenotypic study of 13 cases of *de novo* CD5^+^ DLBCL showed heterogeneous features [23]. No large-scale study of CD5^+^ DLBCL in Western countries has been performed with attention focused on the clinicopathological features and clinical response to R-CHOP.

Biological study of CD5^+^ DLBCL can enhance understanding of the pathogenesis. Recent gene expression profiling (GEP) analysis by two groups yielded contradictory results. In one study comparison of 11 *de novo* CD5^+^ DLBCL and 9 CD5^−^ DLBCL cases showed upregulation of integrin-β 1 and/or CD36 adhesion molecules, which were confirmed by immunohistochemistry (IHC) to be expressed in tumor cells and vascular endothelia, respectively [24]. In another study that compared 22 CD5^+^ and 26 CD5^−^ DLBCL cases, CD5 positivity was associated with downregulation of extracellular matrix (ECM)-related genes [25].

The purpose of this study is to assess the frequency, clinicopathologic and biological features of *de novo* CD5^+^ DLBCL and to evaluate the prognostic significance of CD5 expression in DLBCL treated with rituximab-CHOP (R-CHOP) in Western countries.

## RESULTS

### Frequency of CD5 expression in DLBCL and associated clinicopathologic features

**Figure 1:**
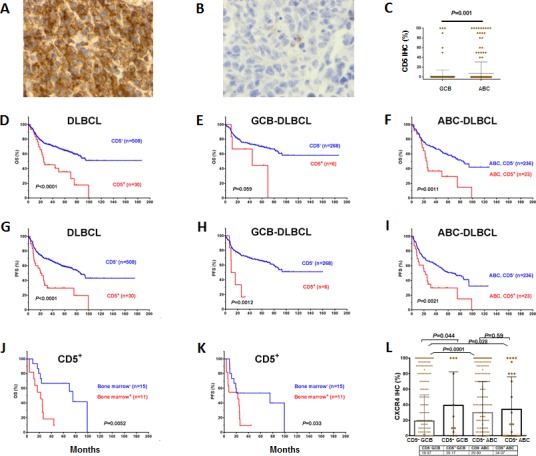
Expression and prognostic significance of CD5 in *de novo* DLBCL (A-B) Representative of CD5^+^ and CD5^−^ immunohistochemical staining; (C) Distribution and comparison of CD5 expression between GCB- and ABC-DLBCL; (D-I) CD5 expression was correlated with significantly poor OS and PFS in the overall, GCB-, and ABC- DLBCL cohorts; (J-K) Bone marrow involvement was correlated with significantly poorer OS and PFS in patients with CD5^+^ DLBCL; (L) Expression of CXCR4 in CD5^+^ and CD5^−^ GCB- or ABC-DLBCL patients.

Figures 1A-B shows representative positive and negative CD5 IHC staining in DLBCL. We observed that thirty (5.6%) DLBCLs in the training set, and eighteen (5.3%) DLBCLs in the validation set were CD5 positive DLBCL. Expression of CD5 was noted on most tumor cells of CD5^+^ DLBCL; 67% of CD5^+^ tumors had >80% of the tumor cells positive for CD5. Most (76.7%) CD5^+^ DLBCL patients were of activated B-cell–like (ABC) subtype (Figure 1C). CD5^+^ patients DLBCL had significantly higher *CD5* mRNA levels compared to CD5^−^ DLBCL patients (*P* = .0019, Supplemental Figures 1A-B).

Comparison of the clinical characteristics of CD5^+^ *vs.* CD5^−^ DLBCL patients in the training set showed that CD5^+^ DLBCL patients were more frequently elderly (>60 years), and had B-symptoms, high performance status, an IPI score >2, and BM involvement (Table 1). None of the CD5^+^ patients, compared to eight (1.6%) CD5^−^ DLBCL patients, showed CNS involvement at diagnosis. However, four (8.3%) CD5^+^ DLBCL patients had CNS relapse during follow-ups.

**Table 1:** Clinical features of patients between CD5^+^ and CD5^−^ *de novo* DLBCL, and patients between CD5^+^ and CD5^−^ ABC-DLBCL in the training set

Variables	CD5^+^	CD5^−^	CD5^+^ *vs*. CD5^−^	CD5^+^ ABC	CD5^−^ ABC	CD5^+^ ABC *vs*. CD5^−^ ABC
	N(%)	N(%)		N(%)	N(%)	
**Age, years**						
< 60	7(23.3)	225(44.3)	**.024**	5(21.7)	83(35.2)	.19
≥ 60	23(76.7)	283(55.7)		18(78.3)	153(64.8)	
**Sex**						
F	14(45.2)	210(41.3)	.67	10(43.5)	96(40.7)	.79
M	17(54.8)	298(58.7)		13(56.5)	140(59.3)	
**Stage**						
I - II	9(32.1)	235(47.8)	.11	7(31.8)	91(39.6)	.48
III - IV	19(67.9)	257(52.2)		15(68.2)	139(60.4)	
**B-symptoms**						
No	13(46.4)	319(65.9)	**.036**	10(45.5)	137(60.6)	.17
Yes	15(53.6)	165(34.1)		12(54.5)	89(39.4)	
**LDH level**						
Normal	9(32.1)	182(38.8)	.48	7(30.4)	79(36.2)	.58
Elevated	19(67.9)	287(61.2)		16(69.6)	139(63.8)	
**No. of extranodal sites**						
0 - 1	22(76.7)	369(77.2)	.75	17(78.3)	169(74.2)	.99
≥ 2	8(23.3)	117(22.8)		6(21.7)	60(25.8)	
**ECOG performance status**						
0 - 1	17(60.7)	387(85.1)	**.0007**	14(60.9)	179(82.9)	**.01**
≥ 2	11(39.3)	68(14.9)		9(39.1)	37(17.1)	
**Size of largest tumor**						
< 5cm	9(45.0)	224(59.4)	.2	7(43.8)	104(58.1)	.27
≥ 5cm	11(55.0)	153(40.6)		9(56.3)	75(41.9)	
**IPI score**						
0 - 2	13(48.3)	310(63.4)	**.05**	10(43.5)	127(55.2)	.28
3 - 5	16(51.7)	182(36.6)		13(56.5)	103(44.8)	
**Therapy response**						
CR	20(66.7)	396(78)	.15	16(69.6)	192(78)	.35
PR	6	64		5	34	
SD	2	20		0	9	
PD	2	28		2	11	
**BM involvement**						
Yes	11(42.3)	39(8.9)	**<.0001**	9(47.4)	20(9.7)	**.0001**
No	15(57.7)	399(91.1)		10(52.6)	187(90.3)	
**COO**
GCB	7(23.3)	268(53.2)	**.002**			
ABC	23(76.7)	236(46.8)				

Note: LDH, serum lactate dehydrogenase; IPI, International Prognostic Index; BM, bone marrow; COO, cell-or-origin; GCB, germinal center B-like; ABC, activated B-cell like.

Pathological features of CD5^+^ *vs.* CD5^−^ DLBCL patients were characterized by comparing their protein expression profiles (Table 2 showed the results for most but not all the biomarkers in 879 patients). CD5^+^ DLBCL, as compared with CD5^−^ DLBCL, were more often positive for Bcl-2, FOXP1, pSTAT3, c-Rel and CXCR4, and less often expressed GCET, CD10, CD30, and SSBP2 (single-stranded DNA binding protein 2), or had *MYC* nonsilent mutations (Table 2, Supplemental Figures 1C-E). *REL* amplification or *BCL2* translocation, which was found in only one CD5^+^ GCB-DLBCL DLBCL, did not account for the increased c-Rel or Bcl-2 level. CD5^+^ DLBCL also had no association with *BCL2* amplifications, unlike one earlier study [26]. When comparison was restricted to the ABC subtype, CD5^+^ DLBCL were associated with significantly higher frequencies of Bcl-2^+^ and pSTAT3^+^ and lower frequencies of *MYC* mutations, CD30^+^, SSBP2^+^, and NF-κB1/p50^+^ (Table 2).

**Table 2:** Pathological features of patients with CD5^+^ and CD5^−^ *de novo* DLBCL (combined training and validation sets)

Variables	CD5^+^	CD5^−^	CD5^+^ vs. CD5^−^	CD5^+^ ABC	CD5^−^ ABC	CD5^+^ ABC vs. CD5^−^ ABC
	N(%)	N(%)		N(%)	N(%)	
**Bcl-2 overexpression**						
< 70%	12(25.5)	369(47.2)	**.0039**	5(15.2)	145(39.2)	**.0076**
≥ 70%	35(74.5)	412(52.8)		28(84.8)	225(60.8)	
***BCL2* gene**						
Normal	31(83.8)	520(78.4)	.13	19(79.2)	248(84.3)	0.38
Translocation	1(2.7)	86(13)		0(0)	9(3.1)	
Amplification or polysomy	5(13.5)	57(8.6)		5(20.8)	37(12.6)	
***MYC* nonsilent CDS mutations**						
Mut	1(2.6)	97(17.5)	**.04**	0(0)	39(15.2)	**.034**
WT	38(97.4)	458(82.5)		27(100)	218(84.8)	
**CXCR4 expression**						
< 20%	21(52.5)	461(66)	**.089**	15(53.6)	194(59.1)	.56
≥ 20%	19(47.5)	237(34)		13(46.4)	134(40.9)	
**GCET overexpression**						
< 50%	40(85.1)	577(70.8)	**.044**	31(93.9)	339(87.8)	.40
≥ 50%	7(14.9)	238(29.2)		2(6.1)	47(12.2)	
**FOXP1 overexpression**						
< 60%	13(27.7)	352(43.2)	**.047**	4(12.1)	78(20.1)	.36
≥ 60%	34(72.3)	463(56.8)		29(87.9)	310(79.9)	
**CD10 overexpression**						
< 40%	34(59.6)	518(63)	.67	28(84.8)	375(95.9)	**.017**
≥ 40%	23(40.4)	304(37)		5(15.2)	16(4.1)	
**CD30**						
Negative	44(95.7)	674(81.8)	**.015**	33(100)	311(79.9)	**.0016**
Positive	2(4.3)	150(18.2)		0(0)	78(20.1)	
**Blimp-1 expression**						
< 10%	32(68.1)	492(66.8)	1.0	21(63.6)	205(59.8)	.71
≥ 10%	15(31.9)	244(33.2)		12(36.4)	138(40.2)	
**pStat3 overexpression**						
< 50%	25(61)	611(85.3)	**.0002**	16(55.2)	279(82.1)	**.0014**
≥ 50%	16(39)	105(14.7)		13(44.8)	61(17.9)	
**Cyclin D3**						
Negative	38(90.5)	526(77.5)	**.05**	28(93.3)	248(76.8)	**.037**
Positive	4(9.5)	153(22.5)		2(6.7)	75(23.2)	
**p50 nuclear expression**						
Negative	23(53.5)	337(45.4)	.35	17(54.8)	129(36.6)	**.05**
Positive	20(46.5)	405(54.6)		14(45.2)	223(63.4)	
**p52 nuclear expression**						
Negative	36(80)	537(76)	.59	27(84.4)	254(76.3)	.38
Positive	9(20)	170(24)		5(15.6)	79(23.7)	
**p65 nuclear expression**						
Negative	16(36.4)	376(49.2)	.12	11(35.5)	194(52.9)	**.09**
Positive	28(63.6)	389(50.8)		20(64.5)	173(47.1)	
**RelB nuclear expression**						
Negative	25(96.2)	378(85.3)	.15	19(95)	172(83.5)	.33
Positive	1(3.8)	65(14.7)		1(5)	34(16.5)	
**c-Rel nuclear expression**						
Negative	23(56.1)	521(72.3)	**.033**	18(62.1)	255(74.6)	.19
Positive	18(43.9)	200(27.7)		11(37.9)	87(25.4)	
**SSBP2 expression**						
≤ 5%	36(76.6)	283(37.7)	**< .0001**	27(81.8)	182(52.6)	**.0015**
> 5%	11(23.4)	468(62.3)		6(18.4)	164(47.4)	

Note: LDH, serum lactate dehydrogenase; IPI, International Prognostic Index; BM, bone marrow; COO, cell-of-origin; GCB, germinal center B-like; ABC, activated B-cell like. Due to tissue exhaustion, immunohistochemical staining analysis for some markers was not successful in few cases.

### CD5 expression is associated with significantly poorer survival in DLBCL

CD5^+^ DLBCL patients had significantly poorer overall survival (OS) (median OS: 25.3 months *vs.* not reached, hazard ratio [HR]: 3.87, 95% confidence interval [CI] of rate: 1.99-7.51, *P* < .0001) and progression-free survival (PFS) (median PFS: 21.3 *vs.* 85.8 months, HR: 4.31, 95% CI: 2.26-8.23, *P* < .0001) in the training set, regardless of cell-of-origin (COO) (Figures 1D-I). The 5-year OS rates for patients with CD5^+^ *vs.* CD5^−^ DLBCL were 35.5% *vs.* 64.8%, and the 5-year PFS rates for patients with CD5^+^ *vs.* CD5^−^ DLBCL were 29.6% *vs.* 59%, respectively. Between CD5^+^ patients with GCB- and ABC-DLBCL there was no significant difference in OS or PFS (*P* = .76 for OS, and *P* = .51 for PFS).

BM involvement significantly impacted OS (*P* = .0052) and PFS (*P* = .033) of CD5^+^ DLBCL patients (Figures 1J-K), and CD5 expression appeared to impact nodal DLBCL more than extranodal DLBCL (Supplemental Figures 1F-I) in the training set. CXCR4, a chemokine receptor involved in tumor cell homing to bone marrow and lymph node [27-29] was expressed at higher levels in CD5^+^ compared with CD5^−^ DLBCL (*P*= .05, Supplemental Figure 1E). However, only in GCB-DLBCL was expression of CXCR4 significantly upregulated (*P = .*044). Expression levels of CXCR4 in CD5^+^ were similar to that in CD5^−^ABC-DLBCL (*P = .*59), although significantly higher than in CD5^−^ patients with GCB-DLBCL (*P* = .028, Figure 1L).

### Effect on Bcl-2 expression and prognostic independence of CD5 expression

**Figure 2:**
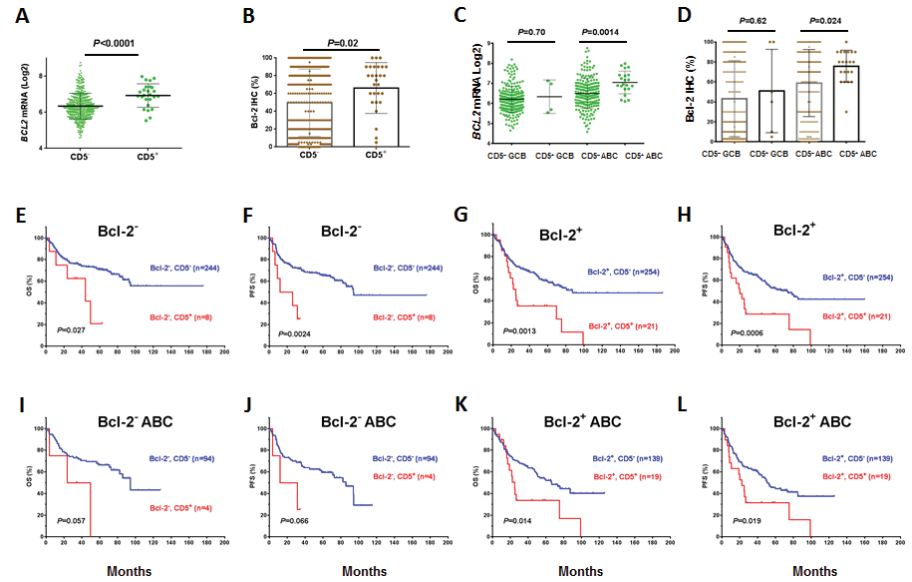
Correlation between CD5 and Bcl-2 overexpression and the prognostic significance of CD5 expression in DLBCL independent of Bcl-2 overexpression (A-D) CD5 expression was correlated with Bcl-2 overexpression and upregulated mRNA levels, in both GCB and ABC subtypes; (E-H) The prognostic significance of CD5^+^ was independent of Bcl-2 overexpression in DLBCL; (I-L) The prognostic significance of CD5^+^ was independent of Bcl-2 overexpression in ABC-DLBCL.

Almost 75% of CD5^+^ DLBCL patients had concurrent overexpression (≥70% of the tumor cells) of antiapoptotic Bcl-2, an unfavorable biomarker [30, 31]. This frequency was significantly higher than that in CD5^−^ DLBCL patients (53%, *P* = .0039, Table 2). Moreover, the correlation of CD5^+^ and Bcl-2 overexpression was observed at both the mRNA and protein levels and remained significant in the comparison of CD5^+^ with CD5^−^ DLBCL within the ABC subtype, which commonly overexpressed Bcl-2 (Figures 2A-D).

However, CD5 expression predicted unfavorable clinical outcomes independent of Bcl-2^+^ status in the overall or ABC-DLBCL (Figures 2E-L), and *vice versa*.

### Effect on NF-κB and STAT3 activation and prognostic independence of CD5 expression

**Figure 3:**
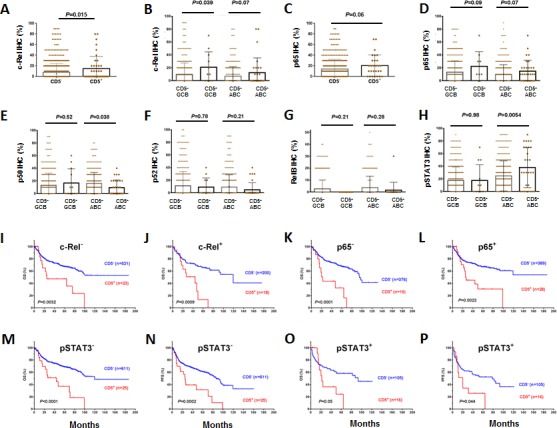
Association between CD5 and NF-κB/STAT3 activation in DLBCL and the independent prognostic significance of CD5 expression (A-D) Association of CD5 expression with nuclear expression of c-Rel and p65; (E) CD5 expression was associated with decreased nuclear expression of p50 in ABC-DLBCL; (F-G) Comparison of nuclear expression of p52 and RelB between CD5^+^ and CD5^−^ patients; (H) CD5 expression was associated with STAT3 activation in ABC-DLBCL; (I-P) The prognostic significance of CD5^+^ was independent of c-Rel, p65 or STAT3 activation.

CD5 expression was associated with nuclear expression of the NF-κB subunits c-Rel and p65, but not mRNA levels of *REL* or *RELA* (Figures 3A-D, Table 2, Supplemental Figures 1J-K). However, nuclear expression of p50, which is also indicative of canonical NF-κB pathway activation [32], was significantly decreased in CD5^+^ ABC-DLBCL (Figure 3E, Table 2), which was not due to the *NFKB1* downregulation at the mRNA level (Supplemental Figure 1L). There was no correlation between CD5^+^ and nuclear expression of p52 or RelB, two subunits involved in noncanonical/alternative NF-κB pathway activation [32] (although showing trends of downregulation in CD5^+^ DLBCL, Figures 3F-G).

CD5^+^ was also associated significantly with nuclear expression of phosphorylated/activated STAT3 but not upreglulated *STAT3* mRNA in the ABC-DLBCL subtype (Figure 3H, Supplemental Figure 1O). The nuclear expression of pSTAT3 has been associated with poorer survival [33].

However, the adverse effect of CD5 expression on prognosis did not depend on c-Rel, p65 or STAT3 activation. In both c-Rel^−^ and c-Rel^+^, p65^−^ and p65^+^, and pSTAT3^−^ and pSTAT3^+^ patients, CD5 expression correlated with significantly poorer OS and PFS (Figures 3I-P, showing analysis in the combined training and validation sets due to the limited CD5+ cases).

### Frequent loss of SSBP2 expression in CD5^+^ DLBCL

**Figure 4:**
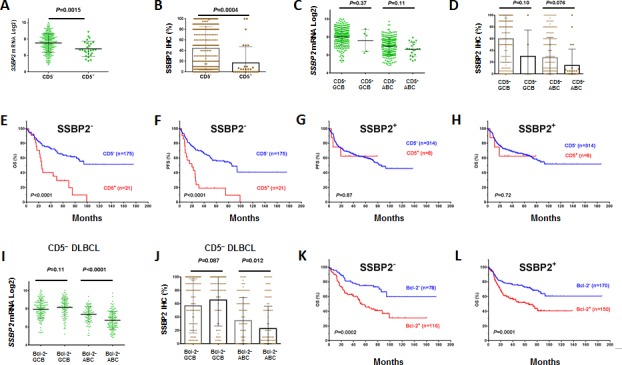
Correlation between CD5 and SSBP2 expression in DLBCL (A-D) CD5 expression was correlated with decreased SSBP2 expression; (E-F) The prognostic significance of CD5^+^ was present only in patients without SSBP2 expression; (G-H) CD5^+^ was not prognostic in patients with SSBP2 expression; (I-J) Bcl-2^+^ compared with Bcl-2^−^ patients had decreased SSBP2 mRNA and protein levels only in the ABC-DLBCL subset but not in the GCB-DLBCL subset; (K-L) The prognostic significance of Bcl-2^+^ was independent of SSBP2 expression.

CD5 positivity in DLBCL was frequently associated with lack of SSBP2 expression, a tumor suppressor protein for lymphoma and leukemia (Figures 4A-D, Table 2) [34-36]. Furthermore, the prognostic significance of CD5 expression was restricted to DLBCLs without or with very low SSBP2 expression. In SSBP2^+^ patients, CD5 expression in DLBCL was not associated with prognosis (Figures 4E-H).

In ABC-DLBCL, Bcl-2^+^ *vs.* Bcl-2^−^ also had decreased SSBP2 mRNA and protein expression independent of CD5 expression (Figures 4I-J, Supplemental Figures 1P-Q). However, the prognostic significance of Bcl-2 was independent of SSBP2 expression (Figures 4K-L).

### Multivariate survival analysis

Multivariate analysis of clinical and pathological factors including IPI (defined by age, stage, serum LDH, performance status, and extranodal sites), sex, B-symptoms and tumor size, CD5^+^, Bcl-2^+^, and COO confirmed that CD5 expression independently predicted significantly poorer OS (*P* = .005) and PFS (*P* = .014) in DLBCL, in addition to the known unfavorable prognostic factors IPI >2 and Bcl-2^+^ (Table 3). COO classification did not reach statistical significance to be an independent factor in this multivariate analysis, but did remain as an independent prognostic factor after removal of CD5 as a factor in the survival analysis, suggesting that CD5 expression significantly contributed to the poor prognosis of ABC-DLBCL.

**Table 3:** Multivariate analysis in the overall and ABC-DLBCL cohort of patients

	OS			PFS		
Variables	HR	95% CI	*p*	HR	95% CI	*p*
**Overall DLBCL**						
**IPI > 2**	2.40	1.70-3.39	**<.0001**	2.24	1.63-3.09	**<.0001**
**CD5^+^**	2.12	1.25-3.5	**.005**	1. 93	1.14-3.24	**.014**
**Bcl-2^+^**	2.04	1.45-2.87	**<.0001**	1.92	1.39-2.63	**<.0001**
**ABC**	1.27	.89-1.81	.19	1.33	.95-1.85	.094
**Female**	.94	.73-1.36	.73	1.0	.67-1.30	.99
**Tumor size>5cm**	1.33	.97-1.83	.078	1.26	.93-1.70	.14
**B-symptoms**	1.27	.90-1.78	.17	1.23	.89-1.70	.20
**ABC-DLBCL**						
**IPI > 2**	2.22	1.44-3.40	**<.0001**	2.08	1.38-3.13	**<.0001**
**CD5^+^**	1. 96	1.06-3.62	**.032**	1. 62	0.88-2.99	.12
**Bcl-2^+^**	1.92	1.26-2.97	**.005**	1.93	1.26-3.88	**.003**
**Female**	1.13	.72-1.78	.58	1.10	0.72-1.70	.64
**Tumor size>5cm**	1.14	.75-1.74	.54	1.05	0.69-1.57	.83
**B-symptoms**	1.14	.72-1.81	.57	1.28	0.82-1.98	.27

Note: OS, overall survival; PFS, progression-free survival; HR, hazard ratio; CI, confidence interval; IPI, International Prognostic Index.

The prognostic significance of these variables (except COO) was further analyzed in the ABC-DLBCL subset. CD5 positivity remained as an independent prognostic factor predicting poorer OS (*P* = .032). However, the *P* value for PFS did not reach statistical significance (*P* = .12). IPI>2 and Bcl-2^+^ remained independent prognostic factors for both OS and PFS (Table 3).

### Prognostic and biological impact of CD5 expression in the validation set

**Figure 5:**
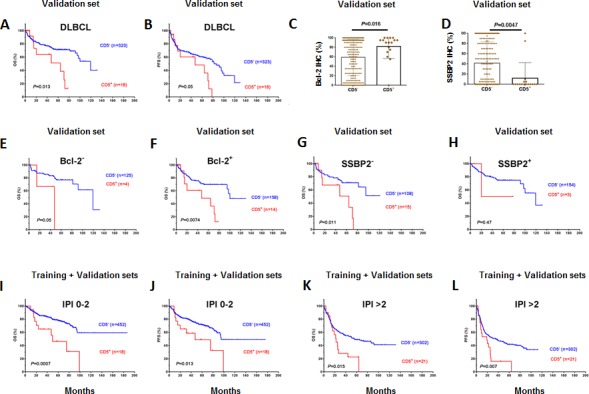
Biological and prognostic impact of CD5 expression in the validation DLBCL cohort (A-B) CD5 expression was correlated with significantly poorer OS and PFS in the overall DLBCL cohort; (C-D) CD5 expression was correlated with Bcl-2 overexpression and decreased SSBP2 expression; (E-F) The prognostic significance of CD5^+^ was independent of Bcl-2 overexpression; (G-H) The prognostic significance of CD5^+^ in the validation DLBCL cohort depended on loss of SSBP2 expression; (I-L) The prognostic significance of CD5^+^ was independent of the IPI.

In an independent validation set from multiple medical centers, we validated the prognostic significance (Figures 5A-B) as well biological impact of CD5^+^ discovered in the training set. In the validation set, CD5^+^ DLBCL were also associated with elevated Bcl-2 and decreased SSBP2 expression (Figures 5C-D), and its prognosis was independent of Bcl-2^+^ (Figures 5E-F) but depended on SSBP2^−^ (Figures 5G-H).

In both the training and validation sets, the prognostic significance of CD5^+^ was independent of the IPI. Figures 5I-L shows analysis in the combined training and validation sets.

### Effect of CD5 expression on BCR/TCR signaling

**Figure 6:**
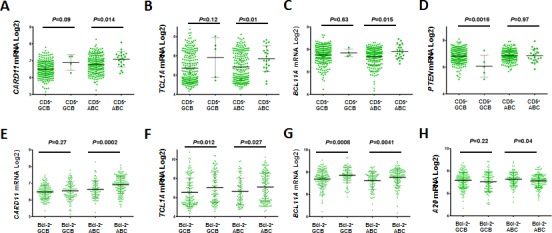
mRNA expression of BCR signaling-related genes between CD5^+^ and CD5^−^ DLBCL patients, or Bcl-2^+^ and Bcl-2^−^ DLBCL patients (A-B) Association of CD5 expression with upregulated *CARD11* and *TCL1* mRNA levels; (C) CD5 expression was associated with increased *BCL11A* mRNA in ABC-DLBCL; (D) CD5 expression was associated with decreased *PTEN* mRNA in GCB-DLBCL; (E-H) Differential expression of *CARD11*, *TCL1, BCL11A* and *A20* between Bcl-2^+^ and Bcl-2^−^ patients resembled the patterns between CD5^+^ and CD5^−^ DLBCL patients.

CD5^+^ DLBCL had significantly higher levels of *CARD11*/*CARM1* mRNA compared with CD5^−^ DLBCL (Figure 6A). CARD11 is a scaffold protein downstream of BCR signaling that activates NF-κB via interaction with BCL10 [32, 37, 38]. *A20*, a negative regulator of NF-κB signaling [37], and *IL10*, which mediates immune inhibition and cell survival downstream the CD5 signaling [10, 11], did not show differential expression of mRNAs between CD5^+^ and CD5^−^ DLBCL patients. (*P*= .30 and *P*= .36). In addition, *TCL1A* (which modulates AKT activation downstream of the TCR signaling [39]) and *BCL11A* (proto-oncogene with a critical role in lymphoid development [40]), which was often co-amplified with *REL* in lymphoid malignancies [41, 42], were significantly upregulated in CD5^+^ ABC-DLBC patients (Figures 6B-C). *PTEN* encoding a tumor suppressor antagonizing the PI3K/AKT signaling was significantly downregulated in CD5^+^ GCB-DLBCL (Figure 6D).

Differential expression of *CARD11*, *TCL1A*, and *BCL11A* between Bcl-2^+^ and Bcl-2^−^ DLBCL patients resembled the effect of CD5 signaling (Figures 6E-G), suggesting that there are similar signaling pathways potentially underlying the association of CD5 and Bcl-2 expression. In contrast, regulation of other genes such as *A20* (Figure 6H), *PTEN*, *CD10*, *PIK3CA*, *MYC*, *CXCL12*, *PRDM1*, *STAT3* showed different patterns in Bcl-2^+^ (*vs.* Bcl-2^−^) and CD5^+^ (*vs.* CD5^−^) patients.

### CD5 expression signatures in *de novo* DLBCL

Gene expression profiles of CD5^+^ and CD5^−^ DLBCL were compared to identify CD5 gene signatures in *de novo* DLBCL, which showed 86 differentially expressed genes (DEGs) by comparison within the overall cohort, and 39 DEGs by comparison within the ABC-DLBCL subcohort (Figures 5A-B, Table 4). Comparison within the GCB-DLBCL subcohort with limited CD5^+^ cases found 17 DEGs, most of which are related to cytoskeleton, microtubule or nervous system function (Figure 5C). Only a few genes were found expressed differentially between CD5^+^ GCB- and ABC-DLBCLs (Table 5).

**Table 4:** Differentially expressed genes between patients with CD5^+^ and CD5^−^ DLBCL

Function categories	Numbers	Upregulated gene names	Numbers	Downregulated gene names
**Antiapoptosis**	2	***BCL2***, *TNFAIP8*		
**T-cell and B-cell receptor signaling**	7	*CARD11, CLECL1, IGHM, LYN, PTPN2, SIT1, SH3BP5*^ⱡ^		
**Hematopoietic development, immune response, central nervous system, signaling including NF-kB, MAPK, Wnt and calcium-dependent pathways**	10	*ARHGAP17, BSPRY, C2orf34*^ⱡ^, ***CXXC5***^ⱡ^, *SIPA1L3*^ⱡ^, *OGT, MPEG1, OAS1*^ⱡ^, TMEM149, GLYR1*	14	*APCDD1*^ⱡ^, *CLEC11A, DZIP1*, ***INHBA***^#x2C61;^, ***PI15***^ⱡ^, PDE4D, **SGK1**, SOBP, CAMK2N1*, FGF1*, FGF17*, PPM1L*, PTPRR*, SLC6A9*
**Cell cycle progression**	3	*CCND2, CDK3, TLK1*	1	*NEK6*^ⱡ^
**Transcriptional activator/repressor**	11	**CREB3L2**, ETV6, FOXP1, IRF2BP2, **JARID2, TCF4**^ⱡ^, ZNF589, RBM39*, ANKRD12*, ZNF614*, ZNF814*	7	LMO2, LMO4, MAML3, TFEC, **MYBL1**, ZNF709, LHX2*
**mRNA stability, RNA regulation**	2	*LUC7L, SERBP1*	1	*SREK1*
**Protein modification, processing, degradation, transporter**	3	*ERP29, FAM175B^ⱡ^, **SLC38A2***	2	*CTSK, UGGT2*^ⱡ^
**Cytoskeletal protein, extracellular matrix, cell adhesion, migration**	1	*PDLIM1*	14	*CCBE1, COL5A1, COL6A3, ENPP3, FAM198B*^ⱡ^, FN1, LTBP1, **PCDH9, PTK2, PTPRB**, RAPH1, BAIAP2L1*, ITGBL1*, MYO7A*
**Metabolism**	1	ST3GAL2*		
**IncRNA, unknown function and others**	12	*C13orf18*^ⱡ^, *CXorf21, FAM129C*^ⱡ^, *LOC100506168, LOC202781, KLHL14, KIAA0040*^ⱡ^, LIC7L, MAPS8, RRP7A, NPIP*, NAPSB*	11	*CRNDE,CCDC144B, C15orf48, FRMD6, GLT1D1, LOC100288271*^ⱡ^, *LOC100506457*^ⱡ^, *LOC100133790*^ⱡ^, LOC440864, KIAA1211, SAMD12, LOC100130815*

Note: Genes marked by

* are those identified in comparison of CD5^+^ *vs.* CD5^−^ ABC-DLBCLs; genes marked by

ⱡ are those identified in both CD5^+^ *vs*. CD5^−^ DLBCL and CD5^+^ *vs*. CD5^−^ ABC-DLBCL. Bolded genes are involved in nervous system development or function.

**Table 5:** Differentially expressed genes between patients with CD5^+^ and CD5^−^ GCB-DLBCL, and between patients with CD5^+^ GCB-DLBCL and CD5^+^ ABC-DLBCL (protein function is from NCBI http://www.ncbi.nlm.nih.gov and UniProtKB http://www.uniprot.org)

	CD5^+^ *vs*. CD5^−^ GCB-DLBCL		CD5^+^ GCB *vs*. CD5^+^ ABC-DLBCL	
Function categories	Up	Down	Up	Down
**Ion-channel**		*CHRNA3, KCNK5,*	*TRPM6*	*KCNJ2*
**Nervous system function**	*SERPINE2, SLC38A2*	*DIO2, SSTR1, ADAM22*	*SERPINE2*	
**Immune response**				*LILRA1*
**Protein folding, transportation**		*UGGT2*		
**Transcription**				*TRPS1*
**Cytoskeleton, microtubule**	*WDR69*	*FGD6, CCIN, MAP6*	*WDR69*	*COL28A1*
**Metabolism**		*CYP1A1, CROT*		*GALT*
**Unknown function**		*KIAA0125, GLT1D1, RBFA*	*C4orf34*	

CD5 expression signatures included both activators and inhibitors of TCR/BCR signaling. Activation of BCR/TCR was suggested by upregulation of *CARD11*, *CLECL1* (encoding a T-cell costimulatory molecule), and *IGHM*. However, inhibition of BCR/TCR and increased threshold for activation in CD5^+^ DLBCL was also suggested by upregulation of *PTPN2* and *SIT1* (which negatively regulate TCR signaling), *LYN* (which has roles in inhibiting BCR), and *SH3BP5* (which inhibits BTK signaling), and downregulation of *PDE4D* (which hydrolyzes c-AMP, thereby removing the c-AMP constraint for TCR) in CD5^+^ DLBCL [43].

DEGs downstream of TCR/BCR also suggested activated BCR signaling with negative feedback in CD5^+^ DLBCL. Activation of the NF-κB, MAPK, and Wnt pathways was indicated by upregulation of *CXXC5* (activating NF-κB and MAPK) and *GLYR1* (activating MAPK) and downregulation of *APCDD1* (negative regulator of Wnt signaling), *PI15* (trypsin inhibitor), *CAMK2N1* (CAMK2 inhibitor), *PPM1L* (dephosphorylating MAPKs), and *PTPRR* (sequester and inhibitor of MAPKs). In turn, antiapoptotic *BCL2* and *TNFAIP8* downstream of the NF-κB pathway were significantly upregulated. On the other hand, inhibition of the calcium-dependent signaling, which is required for the activation of proteins downstream BCR including NF-κB and NFAT [44, 45], was indicated by upregulation of *BSPRY* (inhibiting calcium influx) and downregulation of *SGK1*, which activates ion channels and calcium entry.

CD5 expression signatures also included 18 transcription factors, suggesting distinct transcription programs in CD5^+^ DLBCL patients. Upregulated transcription factors included *CREB3L2* (a transcription activator binding to the cAMP response element), *ETV6* (a transcriptional repressor), *FOXP1* (an essential transcriptional repressor of B-cell development), *IRF2BP2* (a transcription corepressor repressing the NFAT target genes including IL2/IL4), *JARID2* (a transcriptional repressor), *TCF4* (a transcription factor binding to the immunoglobulin enhancer), and *ZNF589* (a transcriptional repressor). Downregulated transcription factors included LIM factors *LMO2*, *LMO4*, and *LHX2* (which have roles in proliferation, differentiation and hematopoietic development), *MAML3* (a transcriptional coactivator for NOTCH proteins), *TFEC* (which binds to the immunoglobulin heavy-chain/*IGH* gene enhancer), and *MYBL1* (a strong transcriptional activator). Altogether it appears that transcription factors that repress TCR/BCR signaling and proliferation outnumbered those that enhance TCR/BCR signaling.

Cell cycle and proliferation genes also appeared under concurrent positive and negative regulations in CD5^+^ DLBCL. Three genes promoting cell cycle progression were upregulated, including *CCND2* (a cyclin important for G1/S transition), *CDK3* (a cyclin-dependent protein kinase involved in G0-G1 and G1-S transitions), and *TLK1* (a kinase involved in the regulation of chromatin assembly), whereas *NEK6* (playing an important role in mitotic cell cycle progression) was downregulated. *JARID2* (which negatively regulates cell proliferation signaling) was upregulated, and *CLEC11A*, *PTK2*, *MYBL1*, and *SGK2* genes (which stimulate proliferation) were downregulated.

Supporting a previous study [25], another distinctive feature of the CD5 expression signatures were the downregulation of genes related to cell adhesion, ECM remodeling, and migration (*CCBE1*, *COL5A1*, *COL6A3*, *ENPP3*, *FAM198B*, *FN1*, *ITGBL1*, *PCDH9*, *PTK2*, *RAPH1*, and *MYO7A*). *LTBP1* (functioning in the assembly, secretion, and targeting of TGFβ1 to ECM), *INHBA* (encoding a TGFβ family member), and *PTPRB* (involved in blood vessel remodeling and angiogenesis) were downregulated in CD5^+^ DLBCL.

Gene set enrichment analysis (GSEA) was performed to enrich the relevant pathways. Downregulated “ECM Receptor Interaction and upregulated “Nitrogen Metabolism” had nominal *P-*values < 1% (.006 and .0039 respectively) although no gene sets were enriched by an FDR threshold of 25% (probably due to the small number of CD5^+^ cases, and the highly heterogeneous nature of CD5^−^ DLBCL patients). In addition, “Focal Adhesion” had a nominal *P*-value of .026 with an FDR of 30%. These results reinforced the notion that downregulation of genes involved in ECM and cell adhesion is a prominent feature of the CD5 expression signature (Figures 5D-F). Pathway analysis by the Ingenuity Pathway Analysis (IPA) software showed CD5 expression signatures were associated with functional networks of Hematopoiesis, Nervous System Development and Function, Cellular Growth and Proliferation (Supplemental Figure 2).

## DISCUSSION

*De novo* CD5^+^ DLBCL is a unique subset of DLBCL [1] and has not been studied on a large scale in Western countries. The current study of 879 patients with *de novo* DLBCL identified 48 (5.5%) CD5^+^ patients, associated with higher frequencies of >1 ECOG performance status, BM involvement, CNS relapse, ABC subtype, Bcl-2^+^, and STAT3 activation whereas with lower frequencies of CD30^+^, SSBP2^+^, and *MYC* mutations. CD5 signaling appears to differentially regulate NF-κB subunits, activating c-Rel and p65 but decreasing p50 activation. Other features associated with CD5^+^ DLBCL in this study, such as elderly age, B-symptoms, IPI, GCET, CD10, FOXP1 and CXCR4, were probably due to the predominance of ABC subtype of CD5^+^ DLBCL patients. Compared with previous studies conducted in Japan, CD5^+^ DLBCL in Western countries had lower prevalence, shared common features of performance status, ABC subtype, Bcl-2 overexpression, BM involvement, and development of CNS recurrence, but lacked features of female predominance, extranodal involvement, elevated serum LDH, and higher disease stage [13-15, 17-19]. These differences may reflect ethnic and genetic variation, the heterogeneity of CD5^+^ DLBCL, the larger number of CD5^+^ DLBCLs (*n* = 109) in the Japanese cohort and cohort-specific features.

We further assessed the prognostic impact of CD5 expression and found that CD5^+^ independently correlated with poorer survival in DLBCL with R-CHOP treatment. Moreover, the 48 CD5^+^ DLBCL patients treated by R-CHOP in this study cohort did not show significant improvement in OS (*P*= .66) or PFS (*P*= .81) compared to the 14 CD5^+^ DLBCL patients treated with CHOP from an independent CHOP-treated DLBCL cohort (results not shown). Furthermore, our attempt to understand the biology of CD5^+^ DLBCL suggested that molecular pathways downstream BCR signaling which promote cell proliferation and survival (such as Bcl-2 [but not Myc] overexpression, and activation of c-Rel, p65, and STAT3) were likely relevant for the pathogenesis of CD5^+^ DLBCL; however, the adverse impact of CD5 expression did not depend on any of these factors alone.

BM involvement also appeared to impact prognosis of CD5^+^ DLBCL significantly in the training set (Figures 1J-K, which however was not confirmed in the validation set), and development of CNS relapse (0% at diagnosis, 8.3% after treatment) was remarkable for CD5^+^ DLBCL. A role of CXCR4/CXCL12 axis in BM involvement and CNS relapse of CD5^+^ DLBCL was suggested by the higher CXCR4 expression in the studied CD5^+^ DLBCL patients (Supplemental Figure 1E) [28, 46]. However, restricting within ABC-DLBCL, CD5^+^ compared to CD5^−^ patients had similar levels of CXCR4 but had a higher incidence of BM involvement (34.5% vs. 7.2%, *P < .*0001, Table 2), suggesting that CXCR4/CXCL12 axis was not sufficient to explain for the BM involvement. A previous study in CLL/SLL also suggested that other factors in addition to the CXCR4/CXCL12 axis may account for marrow infiltration of neoplastic cells [46]. In this study, downregulation of genes involved in ECM and cell adhesion, which was a prominent feature of the CD5^+^ signature revealed by GEP analysis, likely contributed to the BM involvement and development of CNS relapse in CD5^+^ DLBCL.

Interestingly, CD30 and SSBP2 (a tumor-suppressor [34, 36, 47]) expression was frequently negative in CD5^+^ DLBCL patients. SSBP2 has a critical regulatory role in the transcriptional program of hematopoietic stem and progenitor cells *in vivo*, via modulating the abundance and function of multiple transcription cofactors including LIM domain-binding protein 1 (LDB1), LMO, and LHX. In mouse models, loss of SSBP2 resulted in hypoplastic hematopoietic tissues and impaired hematopoiesis and was associated with shortened lifespan and greater susceptibility to B-cell lymphomas [34, 35, 48]. Prognostic significance of CD5 expression in SSBP2^+^ and SSBP2^−^ DLBCL patients also suggests a tumor-suppressor function of SSBP2 for CD5 signaling.

GEP analysis suggested both positive and negative regulation of TCR/BCR in CD5^+^ DLBCL patients, and differential regulation of BCR downstream pathways (activation of NF-κB, MAPK, and Wnt pathways and inhibition of calcium influx). This may suggest the activated but reprogrammed BCR signaling in CD5^+^ DLBCL patients, and the role of CD5 expression in mitigating BCR signaling, and promoting tumor cell survival by previous studies [7-10]. Likewise, both positive and negative regulations of proliferation, growth, and cell cycle were suggested by CD5 expression signatures. Therefore it appears that CD5 signaling contributes to survival yet an anergic-like state of B-cells.

It is also possible that the unique characteristics of the CD5 expression signature in antiapoptosis, proliferation, signaling, and transcription reflects the COO and the differentiation stages of the lymphoma cells. For example, downregulated *LMO2, LMO4*, *LHX2,* and *CLEC11A* as well as upregulated *FOXP1*, *ZNF589, LYN*, and *BCL2* are hematopoietic mediators and have distinct expression patterns during B-cell development. In addition, *IGHM* expressed in naïve B-cells and plasma cells was upregulated in CD5^+^ patients, whereas *MYC* mutations, which may arise from an aberrant hypermutation process in DLBCL [49], were almost absent in CD5^+^ DLBCL patients (only one CD5^+^ GCB-DLBCL case had *MYC* mutations). Previous studies indicated that CD5^+^ DLBCL and CD5^+^ CLL had higher frequencies of germline *vs.* somatically hypermutated *IGHV* genes compared with CD5^−^ DLBCL [50], suggesting the COO of CD5^+^ DLBCL might be distinct from that of CD5^−^ DLBCL and yet similar to that of CD5^+^ CLL [51]. Our collective results of GEP, *MYC* mutations, and Blimp-1 expression (indicating commitment to plasma cell differentiation) [52-54] in CD5^+^ and CD5^−^ DLBCL (Tables 2, 4) suggest that subsets of CD5^+^ DLBCL may originate from pre-GC, memory B-cells, or neoplasms differentiated into plasma cells yet never through GC reaction [60, 61].

In summary, in this study we show that *de novo* CD5^+^ DLBCL, which occurs at a low frequency (5.5%) in Western countries, was associated with unfavorable clinicopathologic variables and with inferior survival following R-CHOP treatment. Although heterogeneity still exists in this disease subset, dysregulated BCR signaling is significantly implicated in lymphoma cell survival and disease dissemination. Bcl-2 inhibitors, STAT3 inhibitors, and therapeutic strategies modulating BCR signaling, tumor microenvironment and cytokine/chemokine axes may help in the management of CD5^+^ DLBCL patients.

**Figure 7:**
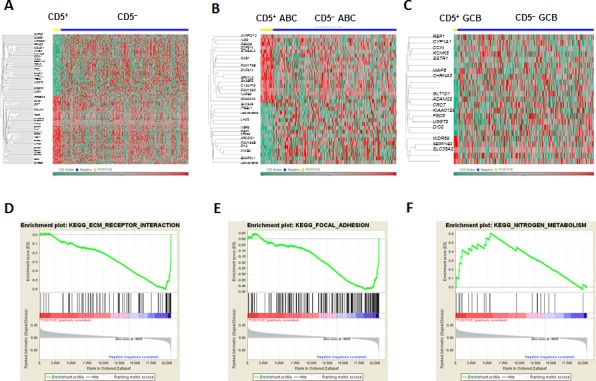
Gene expression profiling and gene set enrichment analysis of CD5DLBCL (A-C) Gene expression profiling of CD5^+^ *vs.* CD5^−^ patients in the overall, ABC-, and GCB -DLBCL cohorts; (D-F) Gene set enrichment analysis of CD5 expression signatures.

## MATERIALS AND METHODS

### Patients

This study was part of the International DLBCL Rituximab-CHOP Consortium Program, comprising a total of 879 patients (538 patients in the training set and 341 patients in the validation set) with *de novo* DLBCL who were treated with R-CHOP chemotherapy. Patients who had a history of low-grade B-cell lymphoma, primary mediastinal, cutaneous, or central nervous system DLBCL, or human immunodeficiency virus infection were excluded. All patients were reviewed by a group of hematopathologists and were diagnosed according to the World Health Organization classification criteria. The study was conducted in accordance with the Declaration of Helsinki, and the protocol was reviewed and approved by the Institutional Review Boards of each participating center, and the comprehensive collaborative study was approved by the Institutional Review Board at The University of Texas MD Anderson Cancer Center.

### Tissue microarray and biomarkers

Immunohistochemical analysis for CD5 expression using a monoclonal CD5 antibody (Novocastra Labs, UK) was performed on 879 DLBCL biopsy specimens using formalin-fixed, paraffin-embedded tissue microarrays as described previously [55, 56]. CD5 expression was scored by three pathologists independently on 400 cells in each of cases under a microscope at 40× magnification. Expression of other markers such as CD10, GCET1, MUM1, FOXP1, Bcl-6, Bcl-2, Myc, Ki-67, p53, MDM2, NF-κB subunits, pSTAT3, CD30, CXCR4, and SSBP2 [31, 33, 55-59] was also assessed using respective antibodies. SSBP2 antibody was kindly provided by Dr. Lalitha Nagarajan, PhD from the Department of Genetics, MD Anderson Cancer Center [34,36]. Due to tissue exhaustion, IHC analysis for some markers other than CD5 was not successful in few cases. Gene translocations and amplifications were detected using the methods described previously [30, 31, 59]. *MYC* mutation was detected using the Sanger sequencing method.

### Cell-of-origin classification

Cell-of-origin classification as either GCB or ABC DLBCLs was determined by GEP for patients in the training set and by IHC according to the Visco-Young algorithm and/or Choi algorithms [55] for all the patients in the training and validation sets.

### Gene expression profiling

GEP were achieved in 488 DLBCL (27 CD5^+^ and 461 CD5^−^) patients of the training set using total RNAs extracted from each formalin-fixed, paraffin-embedded tissue sample and Affymetrix GeneChips array as described previously [30, 55, 57, 58]. The microarray data were quantified and normalized, and the DEGs between CD5^+^ and CD5^−^ DLBCL patients at false discovery rate of .01 were identified using multiple t-tests. Gene set enrichment analysis was performed on the KEGG pathway gene sets. Pathway analysis for the DEGs was performed using the Ingenuity Pathway Analysis software program (IPA, http://www.qiagen.com/ingenuity).

### Statistical analysis

Clinicopathologic differences between different DLBCL subgroups were assessed using Fisher's exact test and the Spearman rank correlation. The mRNA expression levels of affected genes were also retrieved from the GEP data and compared between CD5^+^ and CD5^−^ DLBCL patients using unpaired *t* tests. Overall survival was calculated from the time of diagnosis to death from any cause or last follow-up. Progression-free survival was calculated from the time of diagnosis to disease progression, relapse, or death from any cause. Patients who were alive and/or had no disease progression were censored at last follow-up. Survival analysis was performed using the Kaplan–Meier method with GraphPad Prism 6 (GraphPad Software, San Diego, CA), and differences were compared using the log-rank test. Multivariate survival analysis was performed using the Cox proportional hazards regression model with the SPSS statistics software program (version 19.0; IBM Corporation, Armonk, NY). All differences with *P* ≤ .05 were considered statistically significant.

## SUPPLEMENTARY MATERIAL FIGURES


