# Erratum: Clinical and biological significance of *de novo* CD5+ diffuse large B-cell lymphoma in Western countries

**DOI:** 10.18632/oncotarget.4464

**Published:** 2015-03-08

**Authors:** Zijun Y. Xu-Monette, Meifeng Tu, Kausar J. Jabbar, Xin Cao, Alexandar Tzankov, Carlo Visco, Lalitha Nagarajan, Qinging Cai, Santiago Montes-Moreno, Yuji An, Karen Dybkaer, April Chiu, Attilio Orazi, Youli Zu, Govind Bhagat, Kristy L. Richards, Eric D. Hsi, William WL. Choi, J. Han van Krieken, Jooryung Huh, Maurilio Ponzoni, Andrés J.M. Ferreri, Xiaoying Zhao, Michael B. Møller, John P. Farnen, Jane N. Winter, Miguel A. Piris, Roberto N. Miranda, L. Jeffrey Medeiros, Ken H. Young

**Affiliations:** ^1^ Department of Hematopathology, The University of Texas MD Anderson Cancer Center, Houston, TX, USA; ^2^ Peking University Cancer Hospital and Institute, Beijing, China; ^3^ University Hospital, Basel, Switzerland; ^4^ San Bortolo Hospital, Vicenza, Italy; ^5^ Department of Genetics and Leukemia, University of Texas MD Anderson Cancer Center, Houston, TX, USA; ^6^ Hospital Universitario Marques de Valdecilla, Santander, Spain; ^7^ Aalborg University Hospital, Aalborg, Denmark; ^8^ Memorial Sloan-Kettering Cancer Center, New York, NY, USA; ^9^ Weill Medical College of Cornell University, New York, NY, USA; ^10^ The Methodist Hospital, Houston, TX, USA; ^11^ Columbia University Medical Center and New York Presbyterian Hospital, New York, NY, USA; ^12^ University of North Carolina School of Medicine, Chapel Hill, NC, USA; ^13^ Cleveland Clinic, Cleveland, OH, USA; ^14^ University of Hong Kong Li Ka Shing Faculty of Medicine, Hong Kong, China; ^15^ Radboud University Nijmegen Medical Centre, Nijmegen, Netherlands; ^16^ San Medical Center, Ulsan University College of Medicine, Seoul, Korea; ^17^ San Raffaele H. Scientific Institute, Milan, Italy; ^18^ Zhejiang University School of Medicine, Second University Hospital, Hangzhou, China; ^19^ Odense University Hospital, Odense, Denmark; ^20^ Gundersen Lutheran Health System, La Crosse, WI, USA; ^21^ Feinberg School of Medicine, Northwestern University, Chicago, IL, USA; ^22^ The University of Texas School of Medicine, Graduate School of Biomedical Sciences, Houston, Texas, USA

**Oncotarget. 2015; 8:5615-5633**

PMID: 25760242

Lalitha Nagarajan is added as co-author.

^5^ Department of Genetics and Leukemia, University of Texas MD Anderson Cancer Center, Houston, TX, USA

The corrected list of authors.

Zijun Y. Xu-Monette^1^*, Meifeng Tu^2^*, Kausar J. Jabbar^1^, Xin Cao^1^, Alexandar Tzankov^3^, Carlo Visco^4^, Lalitha Nagarajan^5^, Qinging Cai^1^, Santiago Montes-Moreno^6^, Yuji An^1^, Karen Dybkaer^7^, April Chiu^8^, Attilio Orazi^9^, Youli Zu^10^, Govind Bhagat^11^, Kristy L. Richards^12^, Eric D. Hsi^13^, William WL. Choi^14^, J. Han van Krieken^15^, Jooryung Huh^16^, Maurilio Ponzoni^17^, Andrés J.M. Ferreri^17^, Xiaoying Zhao^18^, Michael B. Møller^19^, John P. Farnen^20^, Jane N. Winter^21^, Miguel A. Piris^6^, Roberto N. Miranda^1^, L. Jeffrey Medeiros^1^, and Ken H. Young^1,22^*

^1^ Department of Hematopathology, The University of Texas MD Anderson Cancer Center, Houston, TX, USA

^2^ Peking University Cancer Hospital and Institute, Beijing, China

^3^ University Hospital, Basel, Switzerland

^4^ San Bortolo Hospital, Vicenza, Italy

^5^ Department of Genetics and Leukemia, University of Texas MD Anderson Cancer Center, Houston, TX, USA

^6^ Hospital Universitario Marques de Valdecilla, Santander, Spain

^7^ Aalborg University Hospital, Aalborg, Denmark

^8^ Memorial Sloan-Kettering Cancer Center, New York, NY, USA

^9^ Weill Medical College of Cornell University, New York, NY, USA

^10^ The Methodist Hospital, Houston, TX, USA

^11^ Columbia University Medical Center and New York Presbyterian Hospital, New York, NY, USA

^12^ University of North Carolina School of Medicine, Chapel Hill, NC, USA

^13^ Cleveland Clinic, Cleveland, OH, USA

^14^ University of Hong Kong Li Ka Shing Faculty of Medicine, Hong Kong, China

^15^ Radboud University Nijmegen Medical Centre, Nijmegen, Netherlands

^16^ San Medical Center, Ulsan University College of Medicine, Seoul, Korea

^17^ San Raffaele H. Scientific Institute, Milan, Italy

^18^ Zhejiang University School of Medicine, Second University Hospital, Hangzhou, China

^19^ Odense University Hospital, Odense, Denmark

^20^ Gundersen Lutheran Health System, La Crosse, WI, USA

^21^ Feinberg School of Medicine, Northwestern University, Chicago, IL, USA

^22^ The University of Texas School of Medicine, Graduate School of Biomedical Sciences, Houston, Texas, USA

* These authors have equal contributions to this work.

