# Supplementary material for: Clinical and biological significance of de novo CD5+ diffuse large B-cell lymphoma in Western countries
Source: Oncotarget. 2015 Mar 8;6(8):5615–33. doi: 10.18632/oncotarget.3479 (PMC4467390; doi:10.18632/oncotarget.3479)
Supplement: Supplementary file 1 [file oncotarget-06-5615-s001.pdf]

Clinical and biological significance of *de novo* CD5$^+$ diffuse large B-cell lymphoma in western countries

Supplementary Material

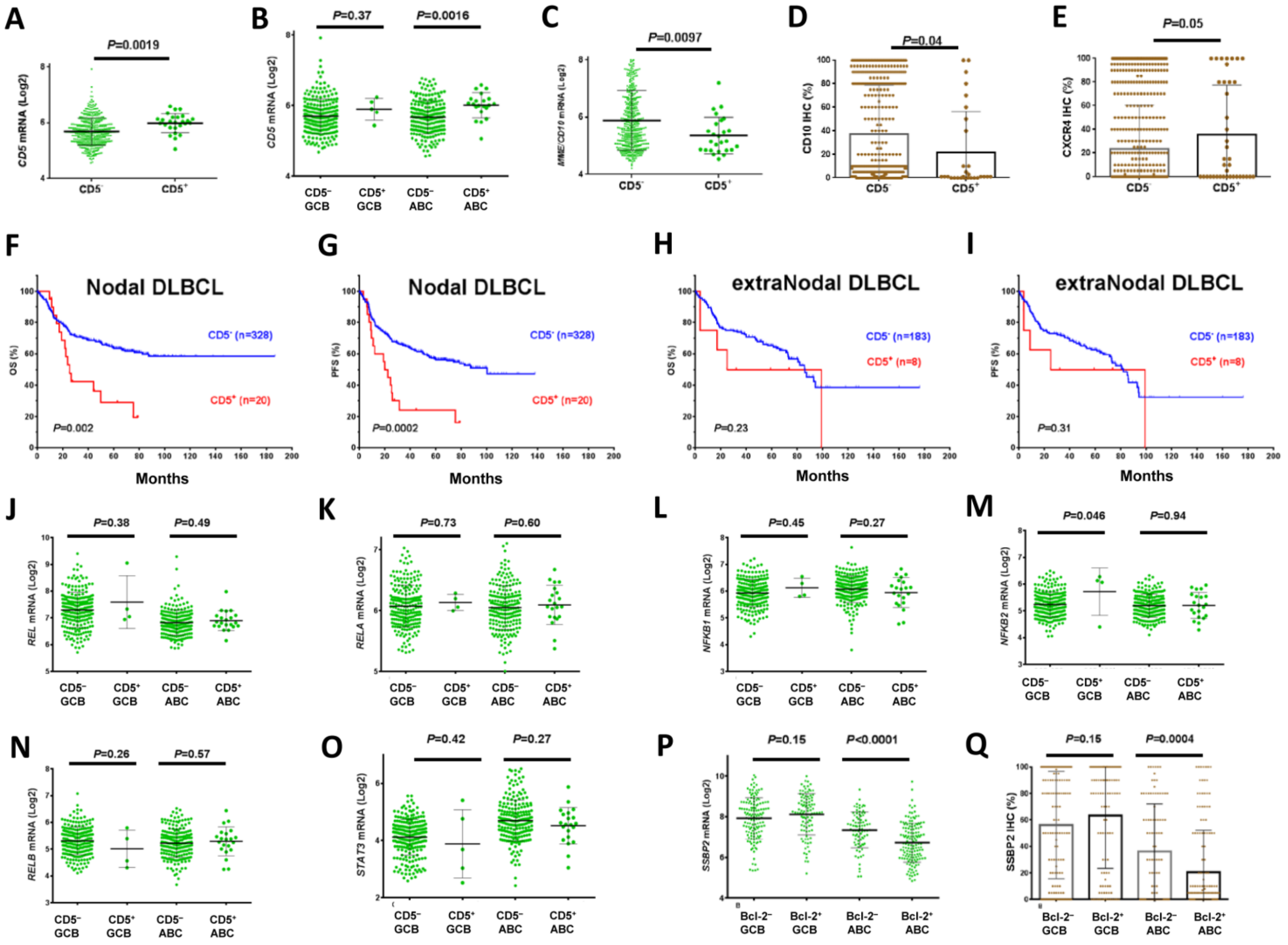

Supplemental Figure 1: (A-B) CD5 expression was correlated with upregulated *CD5* mRNA levels, in both GCB and ABC subtypes. **(C-D)** CD10 expression in CD5$^+$ and CD5$^-$ patients. **(E)** CXCR4 expression in CD5$^+$ and CD5$^-$ DLBCL patients. (**F-I**) The prognostic impact of CD5$^+$ was significant in nodal DLBCL but not in extranodal DLBCL. (**J-O**) CD5 expression did not correlate with *REL*, *RELA*, *NFKB1*, *NFKB2*, *RELB*, or *STAT3* mRNA levels. **(I-J)** Bcl-2 overexpression was correlated with decreased SSBP2 mRNA and protein levels in ABC-DLBCL but not in GCB-DLBCL.

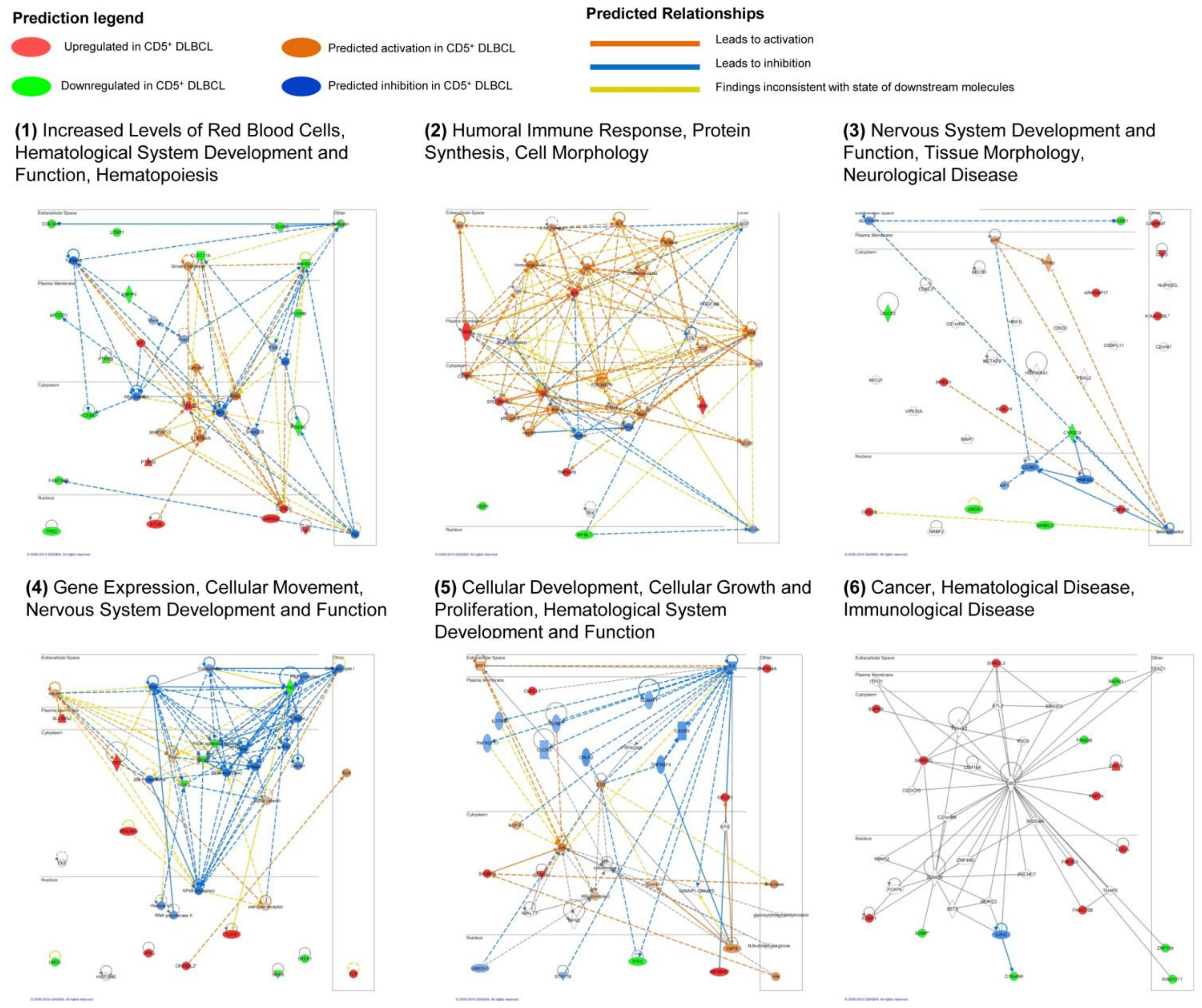

Supplemental Figure 2: Associated networks and pathways showing connections for the CD5 signatures in DLBCL generated by the IPA software (http://www.qiagen.com/ingenuity).

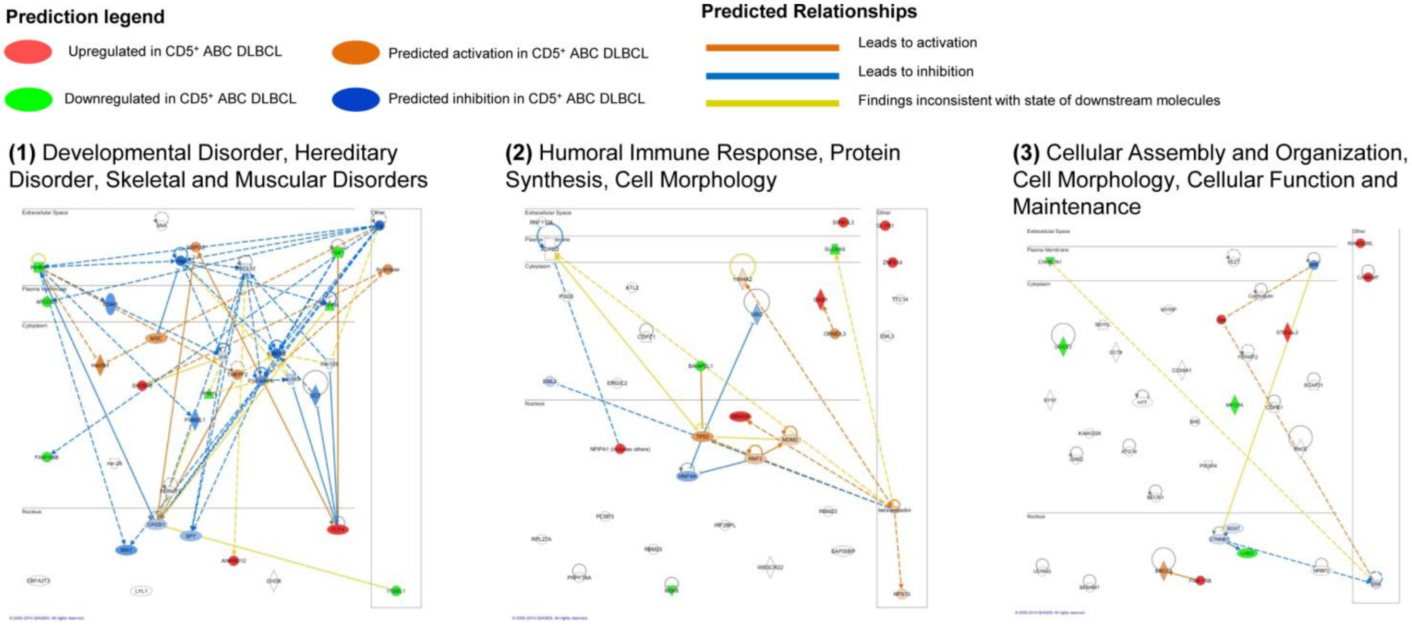

Supplemental Figure 3: Associated networks and pathways showing connections for the CD5 signatures in ABC-DLBCL generated by the IPA software (http://www.qiagen.com/ingenuity).
